# Supplementary material for: Mechanistic Illustration: How Newly-Formed Blood Vessels Stopped by the Mineral Blocks of Bone Substitutes Can Be Avoided by Using Innovative Combined Therapeutics
Source: Biomedicines. 2021 Aug 3;9(8):952. doi: 10.3390/biomedicines9080952 (PMC8394928; doi:10.3390/biomedicines9080952)
Supplement: Supplementary file 1 [file biomedicines-09-00952-s001.zip › biomedicines-1289027-supplementary.pdf]

Supplementary Data

Mechanistic illustration: How newly-formed blood vessels stopped by the mineral blocks of bone substitutes can be avoided by using innovative combined therapeutics

*Author(s), and Corresponding Author(s)**

*Fabien Bornert, François Clauss, Guoqiang Hua, Ysia Idoux-Gillet, Laetitia Keller, Gabriel Fernandez De Grado, Damien Offner, Rana Smaida, Quentin Wagner, Florence Fioretti, Sabine Kuchler-Bopp, Georg Schulz, Wolfgang Wenzel, Luca Gentile, Laurent Risser, Bert Müller, Olivier Huck, Nadia Benkirane-Jessel**

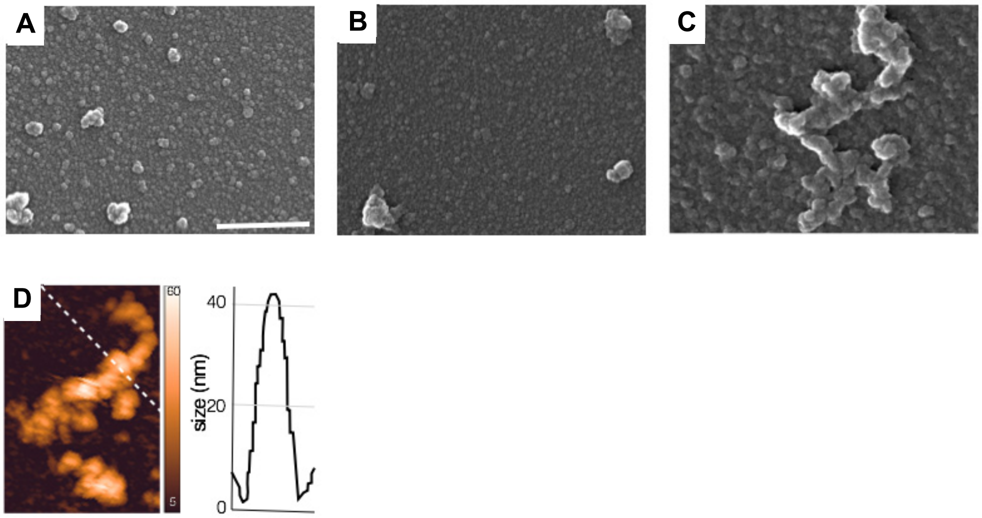

Supplementary Figure S1. Third-generation hybrid bone substitute nano-functionalized with pro-angiogenic molecules. (**A-C**) SEM pictures of VEGF (**A**), HEP (**B**) and HEP/VEGF SNCs (**C**) after deposition on glass slides. Scale bar for A-C: 500 nm. (**D**) The HEP/VEGF complex shown in (**c**), representative of the HEP/VEGF nanocomplexes screened (n = 6), was acquired with an atomic force microscope (AFM). False colors indicate depth, as for the side bar. Measurements taken along the dashed line are plotted in the chart to the right.

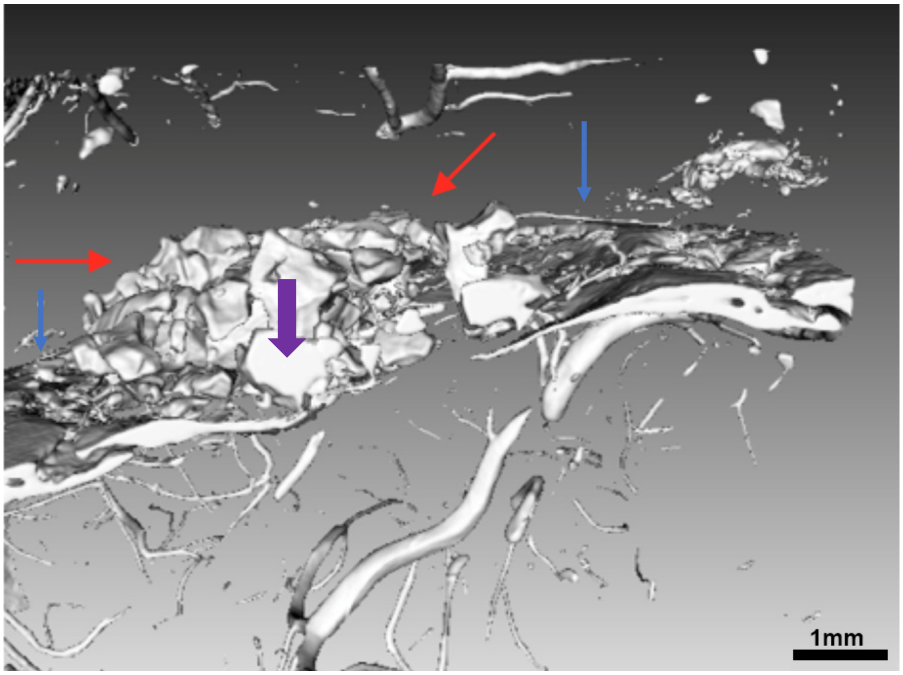

Supplementary Figure S2. Iso-surface 3D reconstruction view of the filled defect before demineralization. Red arrows show hybrid bone substitute (polymeric and mineral blocks), blue arrows show host bone and violet big arrows show mineral blocks.

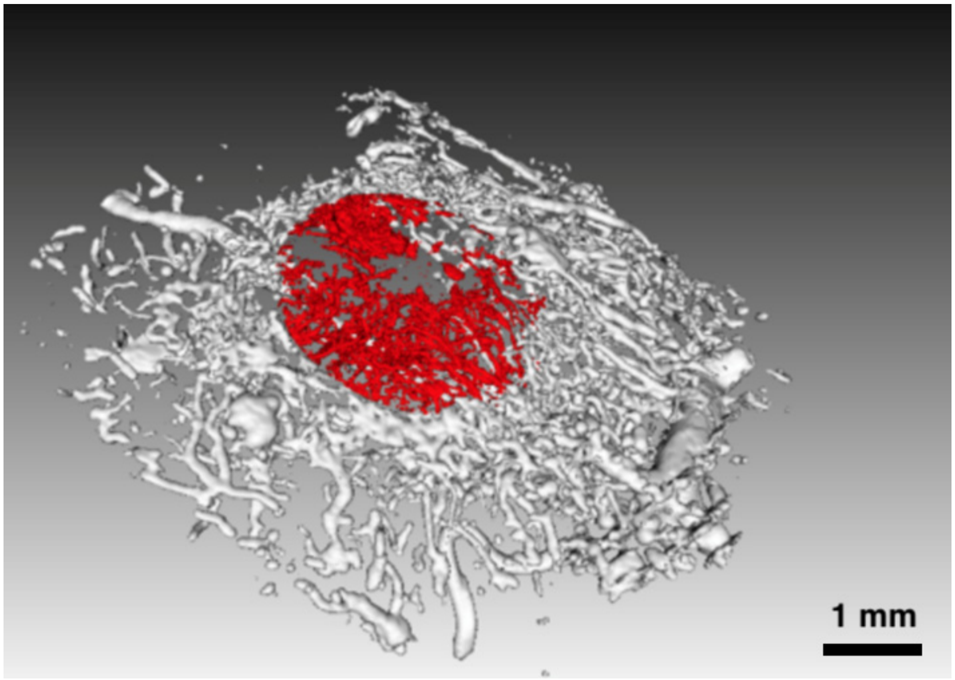

Supplementary Figure S3. Iso-surface 3D reconstruction view of the functionalized hybrid bone substitute after demineralization. Ellipsoid region of interest (ROI) corresponding to bone substitute volume and its neovascularization (in red) showing that no bleeding occurred.

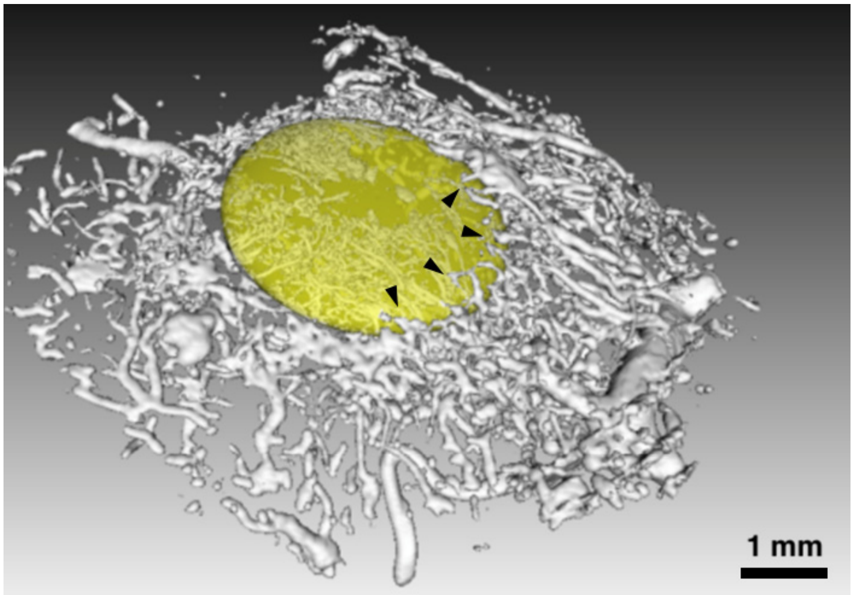

Supplementary Figure S4. Iso-surface 3D reconstruction view of the functionalized hybrid bone substitute after demineralization. Whole view of cranial and bone substitute vascular network (yellow). Black arrowheads indicate the host blood vessels entering into the bone substitute.

Supplementary movie. Bone substitute implanted in a critical size calvarial bone defect at 12 dpi. The animation shows the bone substitute within the calvarial defect and the surrounding tissues, before (0 - 25 sec) and after (26 - 66 sec) demineralization. The host blood vessels become visible within the bone defects after demineralization.
